# Comprehensive expression patterns of inflammatory cytokines in aqueous humor of patients with neovascular age-related macular degeneration

**DOI:** 10.1038/s41598-019-55191-x

**Published:** 2019-12-19

**Authors:** Tomohito Sato, Masaru Takeuchi, Yoko Karasawa, Kei Takayama, Toshio Enoki

**Affiliations:** 1 Department of Ophthalmology, National Defense Medical College, Tokorozawa, Saitama, Japan; 2 Enoki Eye Clinic, Sayama, Saitama, Japan

**Keywords:** Chemokines, Inflammatory diseases

## Abstract

Neovascular age-related macular degeneration (nAMD) is a complex and multi-factorial disease, and low-grade inflammation is associated with pathogenesis of nAMD. Aqueous humor could reflect intraocular immune environments in various eye diseases. The research so far used aqueous humor samples and revealed that inflammation is involved in pathophysiology of nAMD, although immunological roles of cytokines were evaluated inadequately with aspect to individual effects. Here we used 27 kinds of cytokines covering general immunologic reactions, examined specific expression patterns of cytokines, and assessed relationships between inflammation and pathophysiology of nAMD by multivariate analyses. In nAMD eyes, principal component analysis showed that IL-7, MCP-1, MIP-1β and VEGF had high principal component loadings of over 0.6 in the first principal component constituting 32.6% of all variability of the data. In exploratory factor analysis, IL-6, MCP-1 and MIP-1β had high factor loadings (FL) of over 0.5 in Factor 1 constituting 32.6% of all variability, while VEGF had FL of over 1.0 in Factor 3 constituting 10.7% of all variability. In hierarchical cluster analysis, MCP-1 and VEGF were located in the cluster of first proximate mutual distance to central retinal thickness. These data could suggest that low-grade inflammation is a principal contributor in nAMD.

## Introduction

Age-related macular degeneration (AMD) is a leading cause of irreversible blindness in people aged 50 years or older in developed countries^1^. AMD is classified into two forms according to clinical features; dry AMD and neovascular AMD (nAMD)^2^. AMD has complex and multi-factorial etiologies such as age, genetic factors, oxidative stress, inflammation, as well as living environment and lifestyle including smoking^3,4^. AMD is recognized as not only an exudative vascular disease, but also a chronic inflammatory disease in the retina and choroid^1,5^. Several pathogenic pathways have been proposed, such as oxidative stress-induced damage, retinal pigment epithelium cell dysfunction with accumulation of lipofuscin and impairment of lysosomal functions, as well as inflammatory processes with complement activation^6–8^. In particular, systemic and intraocular low-grade inflammations are involved in the pathophysiology of nAMD^9,10^.

Various biological samples have been used to investigate the association of low-grade inflammation with the pathophysiology of AMD. Plasma circulates in the retina and below the retinal pigment epithelium (RPE) which are the main lesions of AMD, and could influence the AMD-associated factors such as immune cells and inflammatory cytokines. Previous studies using human blood samples have indicated that plasma levels of monocyte chemotactic protein-1 (MCP-1) and IL-8 are elevated in nAMD patients, and that monocytes may contribute to the development of nAMD^11–15^. Aqueous humor is also used as a biological sample to reflect intraocular immune conditions. Cytokine levels in the aqueous humor correlate with those in the vitreous fluid, and have been shown to reflect the levels in the vitreous cavity^16,17^. Several previous papers reported the differences in aqueous humor cytokine levels between nAMD patients and cataract patients as controls, and implicated the roles of inflammatory cytokines such as vascular endothelial growth factor (VEGF), interferon gamma-inducible protein 10 (IP-10) and MCP-1 in the pathophysiology of nAMD^9,18,19^. However, these studies evaluated the effects of inflammatory cytokines individually.

Cytokines exhibit multiple and diverse functions by interacting with each other^20,21^. To investigate the effects of inflammatory cytokines involved in complicated immune responses, methods of comprehensive evaluation such as multivariate analysis technique should be used to obtain a global overview of cytokine network patterns.

The purpose of this study was to investigate specific expression patterns of inflammatory cytokines in the aqueous humor, which are associated with the pathophysiology of nAMD.

## Results

### Subjects

According to the classification and diagnostic criteria of AMD^2,22^, 21 eyes (33.8%) in nAMD patients were classified as type 1 nAMD, 15 eyes (24.2%) as type 2 nAMD, 25 eyes (40.3%) as polypoidal choroidal vasculopathy (PCV), and 1 eye (1.6%) as retinal angiomatous proliferation (RAP). Since there was only one case of RAP, comparisons among subtypes of nAMD were conducted in type 1, type 2, and PCV groups only.

The demographics of nAMD patients and controls are summarized in Table 1. There were no significant differences in age and gender between total nAMD patients and controls. The best corrected visual acuity (BCVA) (logMAR VA) was 0.44 ± 0.48 (median 0.30) in nAMD patients and 0.61 ± 0.68 (0.30) in the control group, with no significant difference between two groups (Table 2). The central retinal thickness (CRT) measured using the traditional Early Treatment Diabetic Retinopathy Study (ETDRS) grid^23^ was 306.8 ± 95.5 (276) μm in nAMD patients and 268.4 ± 25.5 (266) μm in controls, also with no significant difference.

**Table 1:** Characteristics of nAMD patients and controls.

Category	nAMD					Control	*P* value	
*N*	Total	Typical		PCV	RAP			
		Type 1	Type 2					
	62 (100%)	21 (34%)	15 (24%)	25 (40%)	1 (2%)	20	Total nAMD vs. Control	Among 4 groups
Age (year)	75^#^ (46–94)^†^	75 (55–88)	77 (48–94)	74 (46–94)	81	72 (55–90)	0.385	0.736
Gender (M/F)	41/21	16/5	9/6	15/10	1/0	8/12	0.070	0.134

Four groups comprised of type 1-, type 2- and PCV-nAMD groups and control group.

The comparison between total nAMD eyes and controls was examined by Mann-Whitney U test. The comparisons among 4 groups were examined by Kruskal-Wallis test. nAMD; neovascular age-related macular degeneration, PCV; polypoidal choroidal vasculopathy, RAP; retinal angiomatous proliferation, *N*; number, M; male, F; female, ^#^; median, ^†^; range.

**Table 2:** Aqueous humor levels of cytokines in nAMD patients and controls.

Category	nAMD				Control						*P* value
*N*	62				20						
	Detectable samples (%)	Value			Detectable samples (%)	Value			Cytokine detection range		
		Median	First quartile	Third quartile		Median	First quartile	Third quartile	Higer limit	Lower limit	
logMAR VA	62 (100)	0.301	0.523	0.155	20 (100)	0.301	0.936	0.172	—	—	0.513
CRT	62 (100)	276	361	243	20 (100)	266	277	251	—	—	0.382
PDGF-BB	0 (0)	0	0	0	0 (0)	0	0	0	22416	1.55	—
IL-1β	0 (0)	0	0	0	0 (0)	0	0	0	7978	0.34	—
IL-1ra	5 (8.06)	0	0	0	0 (0)	0	0	0	95441	5.73	0.509
IL-2	0 (0)	0	0	0	0 (0)	0	0	0	18043	0.67	—
IL-4	0 (0)	0	0	0	1 (5)	0	0	0	4478	0.15	0.523
IL-5	0 (0)	0	0	0	0 (0)	0	0	0	6143	0.61	—
**IL-6**	41 (66.1)	4.52	10.7	0	17 (85)	40.5	145.4	6.00	36654	1.39	**1**.**90** × **10**^−4^**
IL-7	62 (100)	8.54	12.5	5.82	20 (100)	10.9	12.6	7.31	16433	0.64	0.231
IL-8	40 (64.5)	5.68	11.9	0	13 (65)	5.72	9.68	0	28304	1.44	0.535
IL-9	1 (1.61)	0	0	0	1 (5)	0	0	0	28980	1.43	0.531
IL-10	0 (0)	0	0	0	0 (0)	0	0	0	27318	2.14	—
IL-12	39 (62.9)	7.60	13.9	0	15 (75)	9.80	13.3	1.14	34353	2.38	0.402
IL-13	39 (62.9)	1.43	3.03	0	12 (60)	1.61	2.91	0	8160	0.36	0.532
IL-15	0 (0)	0.30	0.52	0.15	0 (0)	0	0	0	20231	1.38	—
IL-17A	0 (0)	0	0	0	0 (0)	0	0	0	32486	2.17	—
Eotaxin	24 (38.7)	0	5.88	0	4 (20)	0	1.13	0	25274	1.26	0.268
bFGF	0 (0)	0	0	0	1 (5)	0	0	0	4069	0.85	0.523
G-CSF	3 (4.84)	0	0	0	1 (5)	0	0	0	39235	1.77	0.546
GM-CSF	0 (0)	0	0	0	0 (0)	0	0	0	13121	3.01	—
IFN-γ	1 (1.61)	0	0	0	0 (0)	0	0	0	25411	3.35	0.539
**IP-10**	62 (100)	426.3	0	321.7	20 (100)	245.2	344.5	81.6	34038	5.18	**9**.**57** × **10**^−5^**
MCP-1	62 (100)	125.4	191.7	101.5	20 (100)	227.5	273.0	87.4	13389	1.29	0.268
MIP-1α	16 (25.8)	0	0.85	0	5 (25)	0	0.96	0	1064	0.28	0.538
MIP-1β	62 (100)	27.3	47.1	17.2	20 (100)	29.2	49.9	19.1	12973	0.28	0.457
RANTES	0(0)	0	0	0	0(0)	0	0	0	4046	1.14	—
TNFα	0(0)	0	0	0	0 (0)	0	0	0	59069	3.25	—
VEGF	62 (100)	110.5	171.2	54.7	20 (100)	121.6	202.2	94.1	38764	1.63	0.182

Cytokine values are given in units of pg/mL. Cytokine levels were compared between nAMD eyes and controls by Mann-Whitney U test. CRT; central retinal thickness, PDGF-BB; platelet derived growth factor BB, IL-1ra; IL-1 receptor antagonist, bFGF; basic fibroblast growth factor, G-CSF; granulocyte colony-stimulating factor, GM-CSF; granulocyte-macrophage colony-stimulating factor, IP-10; interferon gamma-induced protein 10, MCP-1; monocyte chemoattractant protein-1, MIP-1α; macrophage inflammatory protein-1α, RANTES; regulated on activation, normal T-cell expressed and secreted, TNFα; tumor necrosis factor-α, VEGF; vascular endothelial growth factor. **P < 0.01.

### Aqueous humor levels of cytokines in nAMD patients and controls

The profiles of aqueous humor levels of cytokines in nAMD patients before initiation of anti-VEGF agents and in controls are shown in Table 2. Among all the cytokines measured, IL-6 level was significantly lower (*P* = 9.34 × 10^−5^, Cohen’s d = 1.44) but IP-10 level was significantly higher (*P* = 4.80 × 10^−5^, Cohen’s d = 0.71) in total nAMD patients compared to controls, while the other cytokine levels were not significantly different between 2 groups.

When logMAR VA, CRT and cytokine levels were compared among subtypes of nAMD and controls (Table 3), CRT was significantly higher (*P* = 0.007) in type 2 group than that in control group. The levels of IL-6 and IP-10 in all groups of nAMD subtypes were significantly lower and higher than those in control group. Furthermore, IL-7 level was significantly lower (*P* = 0.036) in PCV group than that in control group. When comparing among nAMD subtypes, logMAR VA and CRT were significantly higher in type 2 group than those in PCV group (*P* = 0.004 and *P* = 0.002, respectively), and logMAR VA was significant higher in type 2 group than that in type 1 group (*P* = 0.005).

**Table 3:** Comparisons of cytokine levels in aqueous humor among nAMD subtypes and controls.

Category	nAMD			Control	*P* value						
Subtype	Type 1	Type 2	PCV	20	Among 4 groups	Control vs.			Type 1 vs.		Type 2 vs.
*N*	21	15	25			Type 1	Type 2	PCV	Type 2	PCV	PCV
Value	Median										
**logMAR VA**	0.222	1.046	0.222	0.301	0.004**	0.646	0.292	0.574	0.005**	1.000	0.004**
**CRT**	259	395	260	266	0.002**	0.999	0.007**	0.972	0.051	0.698	0.002**
Gender (M/F)	16/5	9/6	15/10	8/12	0.134	0.042*	0.407	0.301	0.501	0.395	0.739
PDGF-BB	0	0	0	0	—	—	—	—	—	—	—
IL-1β	0	0	0	0	—	—	—	—	—	—	—
IL-1ra	0	0	0	0	0.404	0.761	0.344	0.573	0.764	0.961	0.950
IL-2	0	0	0	0	—	—	—	—	—	—	—
IL-4	0	0	0	0	0.384	0.733	0.821	0.676	—	—	—
IL-5	0	0	0	0	—	—	—	—	—	—	—
**IL-6**	4.34	9.24	4.27	40.5	0.001**	0.006**	0.046*	0.002**	0.777	0.994	0.673
**IL-7**	12.0	10.2	7.97	10.9	0.031*	0.995	0.989	0.036*	0.999	0.175	0.105
IL-8	5.68	6.19	5.29	5.72	0.759	0.872	0.976	0.971	0.999	0.767	0.936
IL-9	0	0	0	—	0.427	0.733	0.995	0.676	0.635	—	0.566
IL-10	0	0	0	0	—	—	—	—	—	—	—
IL-12	9.22	10.6	0	9.80	0.170	0.961	0.985	0.273	0.807	0.655	0.178
IL-13	1.54	2.86	1.11	1.61	0.283	0.987	0.538	0.852	0.880	0.791	0.204
IL-15	0	0	0.22	0	—	—	—	—	—	—	—
IL-17A	0	0	0	0	—	—	—	—	—	—	—
Eotaxin	0	0	0	0	0.554	0.792	0.643	0.512	0.987	0.976	0.999
bFGF	0	0	0	0	0.384	0.733	0.821	0.676	—	—	—
G-CSF	0	0	0	0	0.506	0.929	0.995	0.676	0.995	0.399	0.566
GM-CSF	0	0	0	0	—	—	—	—	—	—	—
IFN-γ	0	0	0	0	0.524	—	—	0.806	—	0.794	0.865
**IP-10**	430.6	737.6	389.1	245.2	**1.42 × 10**^−4^**	0.012*	0.001**	0.003**	0.329	1.000	0.388
MCP-1	121.2	170.2	131.2	227.5	0.315	0.574	0.997	0.573	0.479	0.989	0.578
MIP-1α	0	0	0	0	0.870	0.992	0.982	0.993	0.907	1.000	0.836
MIP-1β	25.6	32.1	28.0	29.2	0.805	0.953	0.987	0.825	0.983	0.998	0.841
RANTES	0	0	0	0	—	—	—	—	—	—	—
TNFα	0	0	0	0	—	—	—	—	—	—	—
VEGF	118.5	153.2	74.2	121.6	0.071	0.796	0.941	0.068	0.759	0.637	0.187

LogMAR VA, CRT and cytokine levels were compared among 4 groups by Kruskal-Wallis test followed by post-hoc Steel–Dwass test for each comparison. *P < 0.05, **P < 0.01.

When gender ratio (proportion of males) was compared among subtype groups of nAMD and control group, a significant difference was observed between type 1 nAMD group and control group (Table 3 and Supplementary Tables S1). Therefore, we conducted a sub-analysis to examine whether this difference affects the other results. When age, logMAR VA, CRT and cytokine levels stratified by gender were compared between type 1 nAMD group and control group (Supplementary Tables S1 and Table S2), IL-6 level was significantly lower (*P* = 0.001) in type 1-male group than that in control-female group, IL-8 level was significantly higher in type 1-female group than those in type 1-male group (*P* = 0.026) and control-female group (*P* = 0.037), and IP-10 level was significantly higher (*P* = 0.008) in type 1-male group than that in control-female group.

### Binomial logistic regression analysis of aqueous humor cytokines related to pathogenesis of nAMD

Since IL-6, IL-7, IL-8, IL-12, IL-13, Eotaxin, IP-10, MCP-1, MIP-1β and VEGF were inflammatory cytokines with detection rates of over 30% in nAMD patients (Table 2), these cytokines were used as explanatory variables in binomial logistic regression analysis, principal component analysis (PCA), exploratory factor analysis (EFA) and multiple regression analysis.

**Figure 1:**
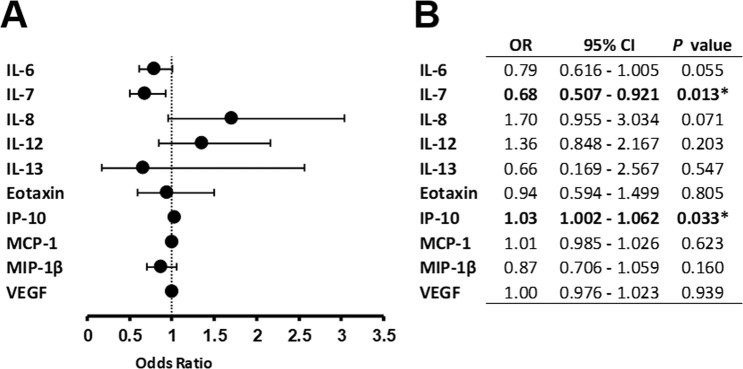
Binomial logistic regression analysis of the contribution of aqueous humor cytokines to pathogenesis of nAMD. Risk factors of developing nAMD are shown in forest plot (**A**) and summary table (**B**) by binomial logistic regression analysis. CI; confidence interval, OR; odds ratio, *P < 0.05.

Binomial logistic regression analysis was performed to examine whether inflammatory cytokines were associated with the risk of developing nAMD (Fig. 1). IP-10 showed a positive association [odds ratio (OR); 1.030, 95% confidence interval (CI); 1.002–1.062, *P* = 0.033], and IL-7 showed a negative association (OR; 0.683, 95% CI; 0.507–0.921, *P* = 0.013) with the development of nAMD. On the other hand, there was no significant association between the remaining inflammatory cytokines and the pathogenesis of nAMD.

### Expression patterns of aqueous humor cytokines by principal component analysis in nAMD patients and controls

**Figure 2:**
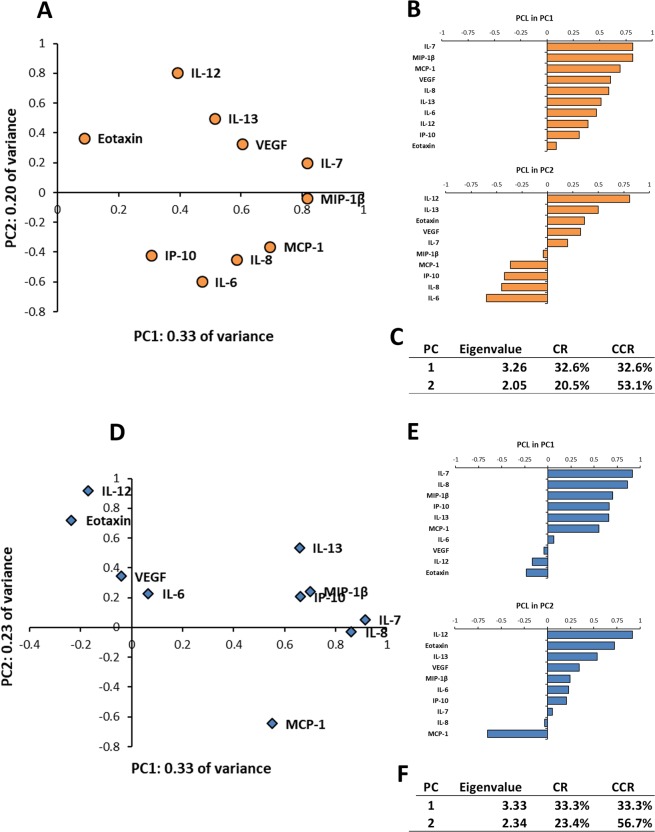
Expression patterns of aqueous humor cytokines by principal component analysis in nAMD patients and controls. Biplots of principal component loading (PCL) in first principle component (PC1) and second principle component (PC2) of nAMD patients (**A**) or controls (**D**) are presented. PCLs of the PC1 and PC2 in nAMD patients (**B**) or controls (**E**) are shown. Eigenvalues, contribution rates (CR) and cumulative contribution rates (CCR) of PC1 and PC2 in nAMD patients (**C**) or controls (**F**) are described. CCR; cumulative contribution ratio, CR; contribution ratio, PC; principle component, PCL; principal component loading, PC1; first principle component, PC2; second principle component.

PCA was performed to express multivariate data simply by converting a set of many correlated variables into a set of fewer uncorrelated variables^24^. In nAMD patients, the eigenvalue of first principal component (PC1) was 3.26, and the PC1 accounted for 32.6% of all variability in the data (Fig. 2). The eigenvalue of second principal component (PC2) was 2.05, and PC2 accounted for 20.5% of all variability in the data. The cumulative contribution rate of PC1 and PC2 was 53.1%. In principal component loading (PCL) analysis of the PC1, all the inflammatory cytokines had positive PCLs, and IL-7, MCP-1, MIP-1β and VEGF had PCLs of 0.6 or above. In particular, IL-7 and MIP-1β had high PCLs of over 0.8. Compared with controls, the PCLs of IL-6, IL-12 and VEGF were elevated remarkably in nAMD patients. In PCL scatter plot of nAMD patients, IL-12, IL-13 and VEGF formed an inflammatory cytokine group, while IL-6, IL-8, IP-10 and MCP-1 formed another inflammatory cytokine group, and the two groups probably work in opposite interaction with each other in the PC2.

### Expression patterns of aqueous humor cytokines by exploratory factor analysis in nAMD patients

**Figure 3:**
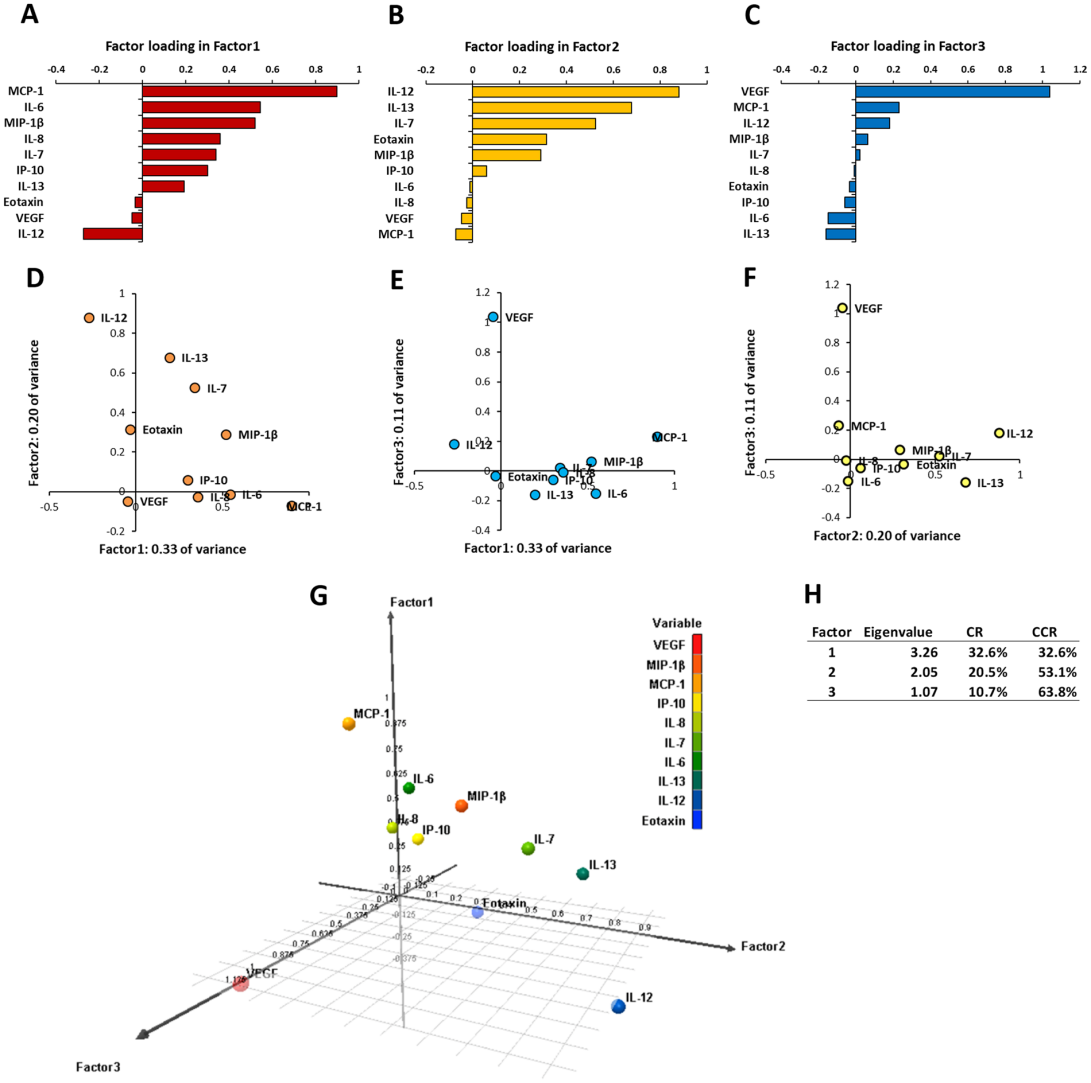
Expression patterns of aqueous humor cytokines by exploratory factor analysis in nAMD patients. (**A**–**C**) Factor loadings (FL) of Factor1 (F1), Factor2 (F2) and Factor3 (F3) in nAMD eyes are drawn. (**D**–**F**) Biplots of FLs in F1, F2 and F3 are presented. (**G**) 3D-plots of FLs in F1, F2 and F3 are shown. (H) Eigenvalues, contribution rates and cumulative contribution rates of F1, F2 and F3 are described.

EFA was performed to identify the underlying relationship between the variable and the phenomenon^25^. In nAMD patients, the eigenvalue of Factor1 (F1) was 3.26, and F1 accounted for 32.6% of all variability in the data (Fig. 3). The eigenvalue of Factor 2 (F2) was 2.05, and F2 accounted for 20.5% of all variability in the data. The eigenvalue of Factor 3 (F3) was 1.07, and F3 accounted for 10.7% of all variability in the data. The cumulative contribution rate of F1, F2 and F3 was 63.8%. In factor loading (FL) analysis of F1, IL-6, MCP-1 and MIP-1β had FLs of 0.5 or above. Especially, MCP-1 had FL of approximately 0.9, and was the cytokine with the strongest influence in F1. On the other hand, IL-12 had FL of -0.27, and was the cytokine with the weakest influence in F1. In FL analysis of F2, IL-7, IL-12, and IL-13 had FLs of 0.5 or above. In particular, IL-12 had FL of approximately 0.9, and was the cytokine with the strongest influence in F2. IL-6, IL-8, MCP-1 and VEGF had minus FLs, and MCP-1 with FL of -0.07 was the cytokine with the weakest influence in F2. In FL analysis of F3, VEGF had FL of over 1.0, and was the cytokine with extremely strong influence in F3, while IL-6 and IL-13 had low FL of approximate -0.15.

### Expression patterns of aqueous humor cytokines by hierarchical cluster analysis in nAMD patients and controls

**Figure 4:**
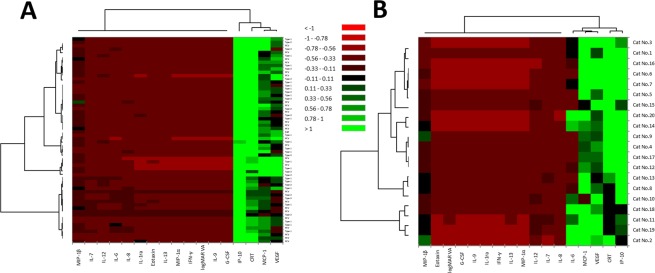
Expression patterns of aqueous humor cytokines by hierarchical cluster analysis in nAMD patients and controls. Heat maps of inflammatory cytokines in aqueous humor of nAMD patients (**A**) and controls (**B**) are presented. Color scale of cytokine levels denotes the following: low cytokine levels, red; middle to high cytokine levels, black to green.

Cluster analysis was performed to classify the inflammatory cytokines, logMAR VA and CRT into relative similar groups called clusters^26^. In nAMD patients, the factors were roughly classified into two principal groups as follows: a high cytokine expression group consisting of IP-10, MCP-1, VEGF and CRT; and a low cytokine expression group consisting of the remaining inflammatory cytokines and logMAR VA (Fig. 4). In the high cytokine expression group, MCP-1 and VEGF were included in the cluster of first proximate mutual distance to CRT, while IP-10 was located in the first proximate mutual distance to the cluster of MCP-1, VEGF and CRT. Regarding the distribution of subtypes of nAMD, type 1, type 2 and PCV groups were not separated clearly in the dendrogram (Fig. 4, vertical axis). In controls, the inflammatory cytokines were also roughly divided into two principal groups as follows: a high cytokine expression group consisting of IL-6, IP-10, MCP-1, VEGF and CRT; and a low cytokine expression group consisting of the remaining inflammatory cytokines and logMAR VA. In the high cytokine expression group, IP-10 was located in the first proximate mutual distance to CRT, while IL-6, MCP-1 and VEGF were included in the cluster of second proximate mutual distance to CRT.

### Correlations among logMAR VA, central retinal thickness and levels of aqueous humor cytokines in nAMD patients

Table 4 show the correlations between the inflammatory cytokines and logMAR VA or CRT in nAMD patients. Multiple linear regression analysis was performed to examine the independent associations of inflammatory cytokines with logMAR VA or CRT^27^. In nAMD patients, logMAR VA correlated positively with CRT and levels of IL-13 and VEGF (*P* = 0.023, *P* = 0.048 and *P* = 0.017, respectively). CRT correlated positively with MCP-1 level (*P* = 0.021), and negatively with VEGF level (*P* = 0.028). When Spearman correlation analyses were conducted to examine correlation among individual cytokines, logMAR VA and CRT, logMAR VA correlated positively with IL-6 level (*P* = 0.037) in nAMD patients, but there was no significant correlation between CRT and the inflammatory cytokines.

**Table 4:** Correlations among best correlated visual acuity, central retinal thickness and levels of aqueous humor cytokines in nAMD patients.

nAMD	Multiple regression analysis											
*N*	62											
**Dependent**		**logMAR VA**						**CRT**				
R		0.584						0.580				
R^2^		0.341						0.336				
**Variables**	**β-coefficient**	**Std. Error**	***t***	***P***		**Variables**	**β-coefficient**	**Std. Error**	***t***	***P***		
**CRT**	0.314	0.0005	2.348	0.023*		**logMAR VA**	0.316	37.65	2.348	0.023*		
IL-6	0.082	0.005	0.563	0.576		IL-6	−0.091	1.272	−0.619	0.538		
IL-7	0.073	0.018	0.404	0.688		IL-7	0.091	5.137	0.501	0.619		
IL-8	−0.023	0.009	−0.145	0.885		IL-8	−0.207	2.494	−1.346	0.184		
IL-12	−0.328	0.015	−1.435	0.157		IL-12	0.237	4.196	1.022	0.312		
**IL-13**	0.326	0.038	2.025	0.048*		IL-13	0.080	11.180	0.479	0.634		
Eotaxin	0.057	0.016	0.456	0.650		Eotaxin	−0.096	4.565	−0.760	0.451		
IP-10	0.034	0.0001	0.251	0.803		IP-10	0.171	0.026	1.287	0.204		
MCP-1	-0.082	0.001	-0.363	0.718		**MCP-1**	0.514	0.219	2.382	0.021*		
MIP-1β	-0.108	0.005	-0.602	0.550		MIP-1β	−0.139	1.351	−0.774	0.443		
**VEGF**	0.489	0.001	2.465	0.017*		**VEGF**	−0.455	0.211	−2.263	0.028*		
**r**_**s**_	**Spearman correlation**											
	**logMAR VA**	**CRT**	**IL-6**	**IL-7**	**IL-8**	**IL-12**	**IL-13**	**Eotaxin**	**IP-10**	**MCP-1**	**MIP-1b**	**VEGF**
logMAR VA	1.000	0.164	0.266	0.207	0.048	0.078	0.130	0.138	0.176	0.186	0.037	0.175
CRT	0.164	1.000	0.006	0.031	−0.085	0.021	0.046	0.003	0.185	0.049	−0.071	−0.108
IL-6	0.266	0.006	1.000	0.381	**0.579**	−0.048	0.028	0.037	**0.558**	**0.513**	0.292	0.080
IL-7	0.207	0.031	0.381	1.000	**0.435**	**0.508**	**0.465**	0.131	0.342	0.214	**0.659**	**0.466**
IL-8	0.048	−0.085	**0.579**	**0.435**	1.000	−0.101	0.071	0.005	0.338	0.389	**0.503**	0.011
IL-12	0.078	0.021	−0.048	**0.508**	−0.101	1.000	**0.573**	0.319	−0.039	−0.313	0.318	**0.680**
IL-13	0.130	0.046	0.028	**0.465**	0.071	**0.573**	1.000	0.096	0.105	−0.312	0.298	0.107
Eotaxin	0.138	0.003	0.037	0.131	0.005	0.319	0.096	1.000	−0.080	0.042	−0.023	0.292
IP-10	0.176	0.185	**0.558**	0.342	0.338	−0.039	0.105	−0.080	1.000	0.357	0.283	0.060
MCP-1	0.186	0.049	**0.513**	0.214	0.389	−0.313	−0.312	0.042	0.357	1.000	0.287	0.190
MIP-1β	0.037	−0.071	0.292	**0.659**	**0.503**	0.318	0.298	−0.023	0.283	0.287	1.000	0.390
VEGF	0.175	−0.108	0.080	**0.466**	0.011	**0.680**	0.107	0.292	0.060	0.190	0.390	1.000
***P*** **value**	**Spearman correlation**											
	**logMAR VA**	**CRT**	**IL-6**	**IL-7**	**IL-8**	**IL-12**	**IL-13**	**Eotaxin**	**IP-10**	**MCP-1**	**MIP-1b**	**VEGF**
logMAR VA	—	0.202	**0.037**	0.107	0.710	0.549	0.315	0.285	0.172	0.148	0.776	0.174
CRT		—	0.962	0.812	0.512	0.871	0.721	0.982	0.151	0.708	0.582	0.402
IL-6	*		—	**0.002**	**8.28 × 10**^**−7**^	0.709	0.826	0.778	**2.51 × 10**^**−6**^	**2.00 × 10**^**−5**^	**0.021**	0.538
IL-7			**	—	4.08 × 10^**−4**^	**2.49 × 10**^**−5**^	**1.42 × 10**^**−4**^	0.310	**0.007**	0.095	**5.75 × 10**^**−9**^	**1.35 × 10**^**−4**^
IL-8			**	**	—	0.437	0.585	0.972	**0.007**	**0.002**	**3.06 × 10**^**−5**^	0.934
IL-12				**		—	**1.13 × 10**^**−6**^	**0.011**	0.766	**0.013**	**0.012**	**1.25 × 10**^**−9**^
IL-13				**		**	—	0.457	0.416	**0.014**	**0.019**	0.408
Eotaxin						*		—	0.535	0.746	0.859	**0.021**
IP-10			**	**	**				—	**0.004**	**0.026**	0.643
MCP-1			**		**	*	*		**	—	**0.024**	0.139
MIP-1β			*	**	**	*	*		*	*	—	**0.002**
VEGF				**		**		*			**	—

BCVA was converted to logMAR units (logMAR VA) for statistical analysis. R; multiple correlation coefficient, R^2^; coefficient of determination, *β*-coefficient; standardized partial regression coefficient, Std. Error; standard error. r_s_; Spearman correlation coefficient, *P < 0.05.

## Discussion

The major findings of the present study are as follows: (1) binomial logistic regression analysis, PCA and EFA revealed that low-grade inflammation is a principal contributor in the development of nAMD, and (2) in hierarchical cluster analysis and multiple regression analysis, MCP-1 was a prominent cytokine associated with CRT which is an essential clinical index of diagnosis and treatment in nAMD.

In this study, CRT and aqueous humor VEGF level did not differ significantly between nAMD patients and controls. Aqueous humor level of VEGF in untreated nAMD eyes varied among previous reports^9,19,28,29^. Furthermore, previous studies have shown no correlation between CRT and aqueous humor VEGF level in untreated nAMD eyes^18,29,30^. Therefore, we suppose that VEGF-driven pathways regulating CNV may be a part of the complex processes in the development of nAMD, and other inflammatory mediators may play a crucial role in deviant angiogenesis^9,18,31^.

In this study, binomial logistic regression analysis identified IL-7 and IP-10 as significant factors for the development of nAMD. IP-10 is a chemokine that attracts type 1 T helper (Th1) cells, and activates Th1 cell-mediated immune response by binding C-X-C motif chemokine receptor 3 (CXCR3)^32,33^. Previous *in vivo* studies found that CC-chemokine receptor 3 and CXCR3 were associated with the development of nAMD^34,35^. In addition, IP-10 functions as an antiangiogenic and antifibrotic factor^36,37^. Bodnar *et al*.^38^ reported that IP-10 inhibited VEGF-induced endothelial cell motility and tube formation *in vitro*. In postmortem eyes with early AMD, IP-10 was strongly expressed in neovascular endothelial cells and connective tissue matrix associated with CNV^39^. Therefore, it is conceivable that IP-10 primarily acts as an antiangiogenic factor to inhibit the development of CNV, and may secondarily induce Th1 cell-mediated immune response to produce low-grade inflammation in nAMD eyes. On the other hand, IL-7 plays a critical role in T cell development and peripheral homeostasis, especially, IL-7 is required for development and survival of regulatory T (Treg) cells^40,41^. Several papers have indicated significant involvement of para-inflammation in the pathophysiology of nAMD, participated by immune cells such as eosinophils, mast cells, macrophages and T cells^9,18,42,43^. Therefore, it is conceivable that IL-7 is a potent mediator of low-grade inflammation, a pathogenetic mechanism of nAMD, via both innate and acquired immune responses.

In this study, IL-6 level was markedly suppressed in nAMD patients compared with controls. IL-6 is both a pro- and an anti-inflammatory cytokine, and induces inflammatory and angiogenic cytokines such as VEGF^44,45^. Agawa *et al*.^9^ reported that aqueous humor IL-6 level in nAMD patients tended to be low compared with that in cataract patients. Therefore, we suppose that down-expression of IL-6 in nAMD eyes may be a part of homeostatic response to suppress CNV development and vascular permeability, although some contradictory results have been reported^46,47^.

PCA is a statistical procedure that uses an orthogonal transformation to convert a set of many correlated variables into a set of fewer uncorrelated variables called principal components^24^. PC1 is the component with primary comprehensive influence on the subject. In this study, the PC1 of nAMD patients was composed of many inflammatory cytokines, and all of those cytokines had positive PCLs. Therefore, these results indicate that inflammatory cytokines have dominant influences on the pathophysiology of nAMD. Especially, IL-7, MCP-1, MIP-1β and VEGF showed high PCLs, suggesting that these inflammatory cytokines and VEGF are responsible factors involved in the pathophysiology of nAMD. MCP-1 is one of the key chemokines regulating migration and infiltration of monocytes/macrophages^48^. Monocytes/macrophages are innate immune cells that digest antigens and present them to other immune cells, enabling them to interact with the adaptive immune system^49^. Previous studies using human aqueous humor reported that MCP-1 is implicated in the pathogenesis of nAMD^9,19,46^. In addition, Lechner *et al*.^14^ have suggested that plasma level of MCP-1 is elevated in nAMD, and monocytes may contribute to the development of nAMD. On the other hand, expression of MIP-1β is increased in the blood of elderly individuals^50,51^. MIP-1β, MCP-1 and IP-10 as well as VEGF in the aqueous humor of nAMD eyes were significantly higher compared with controls^9^. Therefore, based on previous reports and our results, we could assume that inflammation is a principal contributor in the development of nAMD.

In the present study, it is notable that IL-6, IL-12 and VEGF were identified as significant variables with strong influences in the PC1 of nAMD patients, while these cytokines had PCLs of almost 0 in the PC1 of controls. IL-12 activates T cells and NK cells, resulting in the induction of Th1-related immune response^52–54^. In the clinical setting, IL-12 and IL-23 are the major etiologies of psoriasis, psoriatic arthritis and Crohn’s disease^55^. Besides, recent studies indicate that IL-12 has anti-angiogenic effect, and acts as an angiogenic inhibitor in various stages of angiogenesis *in vitro* and *in vivo*^52,56^. Therefore, we presume that IL-12 may primarily play the role of anti-angiogenic factor to inhibit CNV, and secondarily induce Th1-related inflammation associated with the pathophysiology of nAMD. In controls, PCA identified IL-7, IL-8, IL-13, IP-10, MCP-1 and MIP-1β as the principal etiological elements of cataract. However, the cataract patients (controls) were otherwise normal elderly individuals. If the PC1 represents an intraocular immunological environment of the elderly people, the above-mentioned inflammatory cytokines could also be candidates of age-associated inflammatory elements in the elderly eyes.

In PCA, variables with positive PCLs are in conflict with those which have negative PCLs. In the PC2 of nAMD patients, the cytokine group consisting of IL-12 and IL-13 was located on the opposite side of another cytokine group consisting of IL-6, IL-8, IP-10 and MCP-1. In a large scale-cohort study, Ristau *et al*.^57^ suggested that allergy has a protective effect on the development of AMD, although the mechanism remains unclear. Therefore, we would hypothesize that the innate immune responses are in conflict with the adaptive immune responses in nAMD eyes.

EFA is a statistical technique that reduces a large data to a smaller set of summary variables, and identifies underlying relationships between variables and the respondent^25^. In EFA, a factor implies the systematic summary component which is a part of the whole phenomenon, and the influence of the factor depends on the size of the eigenvalue, the same as the contribution rate of the factor. In this study, the F1 was principally composed of IL-6, MCP-1 and MIP-1β, but VEGF had almost no FL in the F1. Besides, the FL of IL-12 was a negative value. In terms of cytokine functions, the F1 may represent the summary component of innate immune response in the pathophysiology of nAMD. The F2 was composed of IL-12, IL-13 and IL-7, but the FL of MCP-1 was below -0.1. Therefore, the F2 may represent the summary component of adaptive immune response. The F3 was composed predominantly of VEGF. Therefore, the F3 would represent the summary component of angiogenic response. Based on the results of EFA, we propose that rather than angiogenic response, inflammatory immune response contributes predominantly in the pathophysiology of nAMD, because the cumulative contribution rate of the F1 and the F2 was 53.1% in the whole pathophysiology of nAMD, while the F3 accounted for only 10.7%.

Cluster analysis is an exploratory method used to classify objects or cases into relative similar groups called clusters^26^. In this study, the cluster related to BCVA was different from that related to CRT. The cluster of cytokines with high expression levels composed of IP-10, MCP-1, VEGF and CRT was common to both nAMD patients and controls. VEGF and MCP-1 are critical cytokines related to vascular permeability^58^. Therefore, VEGF and MCP-1 would be candidates of factors associated with macula edema in nAMD patients as well as elderly individuals. On the other hand, nAMD patients could not be clearly classified into the subtypes of nAMD on the basis of expression levels of inflammatory cytokines (Fig. 4, vertical axis). Therefore, we suppose that there is no significant difference in the specific expression patterns of inflammatory cytokines among the subtypes of nAMD.

Multiple linear regression analysis is a statistical approach used to describe the simultaneous associations of several variables with one continuous outcome^27^. In nAMD eyes, BCVA had a negative correlation with CRT, and MCP-1 was an exacerbation factor of CRT in multiple linear regression analysis. Furthermore, BCVA had a negative correlation with IL-6 level, and IL-6 level showed a positive correlation with MCP-1 level in Spearman’s rank correlation. Jonas *et al*.^30^ reported a positive correlation of MCP-1 level in the aqueous humor with macular thickness in nAMD eyes. On the other hand, VEGF is a key cytokine related to retinal vascular permeability, but previous papers reported no correlation between CRT and aqueous humor VEGF level^18,29,30^. In clinical research, the enrolled nAMD patients had various clinical stages, ranging from early to later clinical stages with macular atrophy^18,59^. Therefore, we suppose that intraocular VEGF levels will fluctuate depending on the clinical stages of nAMD eyes, if strict eligibility criteria for BCVA and macula lesions are not set in the enrollment of nAMD patients.

In exploratory cluster analysis and multiple linear regression analysis, MCP-1 was identified as a critical cytokine associated with CRT of nAMD eyes. This finding suggests that low-grade inflammation affecting macula thickness may be induced by innate immune response via macrophage/monocyte activities in nAMD eyes. Furthermore, BCVA could be improved indirectly depending on the improvement of CRT in nAMD eyes. In the future, combination therapy with anti-VEGF agents and steroids, immunosuppressive drugs and/or anti-TNFα antibodies would be useful to suppress pathological inflammation in nAMD eyes^60–63^.

The present study has several limitations. First, all of the nAMD patients and controls enrolled in this study were Japanese, although it is known that AMD has complex and multi-factorial etiologies including genetic factors, race, high-fat diet and color of the iris^4^. In the future, a large-scale international multicenter study could reveal various immunological features of nAMD depending on the differences in phenotype, genotype, race, as well as living environment and lifestyle. Second, the examination items were limited to 27 inflammatory cytokines, BCVA and CRT as clinical indices. Previous studies reported various nAMD-associated etiological factors other than those examined in our study, including lesion types and lesion size in the macula, insulin-like growth factor-1, angiogenin, monokine induced by interferon γ, soluble intercellular adhesion molecule 1, soluble vascular cell adhesion molecule 1, epithelial growth factor, human growth factor, intercellular adhesion molecule-1, IL-1a2, matrix metalloproteinase 9, plasminogen activator inhibitor 1, and C-reactive protein^9,19,46^. In the future, further multivariate analysis using more variables associated with AMD is needed to provide better understanding of the pathophysiology in AMD. Third, there is a possibility that cytokine levels in aqueous humor are partially influences by cytokine levels in plasma, and are also affected by some unspecified inflammatory substances in the anterior chamber. Fourth, the cytokine levels in aqueous humor are not in full accordance with those in vitreous fluid that is in contact with the impaired retina in nAMD eye. Further studies using various biological samples such as plasma and vitreous fluid together with aqueous humor are warranted to confirm our preliminary results. Fifth, cataract patients were not perfect controls for nAMD patients in this study, because intraocular inflammation in the anterior chamber could contribute to cataract formation. However, performing limbal paracentesis on healthy individuals is not feasible from ethical viewpoint. Therefore, in interpreting the control data, potential increase of inflammatory substances that may influence the levels of aqueous inflammatory cytokines should be considered. Sixth, in analysis of nAMD subtypes, gender ratio was significantly higher in type 1 nAMD group than in control group. However, all multivariate analyses were performed using data of total nAMD patients, and there was no difference in gender ratio between total nAMD patients and controls. Hence, the difference in gender ratio of type 1 nAMD group should not affect the interpretation of results of multivariate analyses.

The strength of the present study was considered that the study population was relatively homogeneous. Fifteen (24.2%) of the enrolled nAMD patients had undergone cataract surgery, and the surgery was performed more than 6 months before this study. Furthermore, the enrolled nAMD patients had not been treated with regular anti-inflammatory eye drops including steroids for postoperative management after cataract surgery. Therefore, we suppose that the aqueous humor samples of nAMD eyes were collected under relatively unbiased conditions, and thus allowed precise evaluation of the specific expression patterns of inflammatory cytokines in nAMD eyes.

In conclusion, the present study indicates that low-grade inflammation via both innate and adaptive immune responses is a principal contributor in the development of nAMD. In the future, we will further examine predictive markers of response to anti-VEGF treatment in nAMD eyes, using comprehensive statistical analyses.

## Methods

### Subjects

This prospective observational study enrolled 62 eyes of 62 nAMD patients and 20 eyes of 20 cataract patients as controls. This study was performed at National Defense Medical College Hospital and Enoki Eye Clinic in Japan between September 1, 2013 and August 1, 2016. The study protocol was approved by the Ethics Committee of National Defense Medical College, and the procedures conformed to the tenets of the Declaration of Helsinki. Informed consent was obtained from all patients before enrolling in this study.

Inclusion criteria for nAMD patients in this study were as follows: (1) previously untreated choroidal neovascularization (CNV); (2) detection of intraretinal edema, subretinal fluid or pigment epithelial detachment by spectral-domain optical coherence tomography (SD-OCT); (3) absence of concurrent ocular diseases in the affected eye, which had compromised or could have compromised vision and ocular conditions. Exclusion criteria were as follows: (1) clinical features suggesting that CNV was secondary to other causes such as pathologic myopia, trauma and hereditary diseases; (2) myopia greater than -6 diopter, or axial length longer than 26 mm; (3) a history of treatments for nAMD including intravitreal drug injection, photodynamic therapy and systemic or topical steroids including sub-tenon injection; (4) previous intraocular surgery, except cataract surgery performed more than 6 months before the date of enrollment. Inclusion criteria for controls in this study were as follows: (1) no current or history of CNV, ocular trauma, diabetic retinopathy, retinal artery occlusion, retinal vein occlusion, ocular tumor, uveitis and other intraocular inflammations; (2) no remarkable drusen and geographic atrophy, (3) myopia less than -6 diopter, and axial length shorter than 26 mm; (4) any previous intraocular surgeries including scleral buckling; (5) previous external ocular surgeries performed within 6 months before the date of enrollment.

The nAMD patients enrolled in this study were different from the cataract patients enrolled as controls. No patient was recruited for both groups.

We performed a priori sample size calculation using the data of our previous study^18^. We found that for a statistical power of 0.80^64^, the sample size for each group required to detect significant differences in levels of aqueous humor cytokines was approximately 20. Therefore, we attempted to recruit around 20 cases each of type 1 nAMD, type 2 nAMD, PCV, RAP and control in this study.

### Diagnostics and treatments

Diagnosis of nAMD was based on a full ophthalmological examination including BCVA test using a decimal chart, intraocular pressure measurement, slit-lamp biomicroscopy, dilated fundus examination, color fundus photography, fundus fluorescein and indocyanine green angiographies, and SD-OCT (Cirrus HD-OCT; Carl Zeiss Meditec, Dublin, CA, USA). Neovascular AMD was classified according to the classification and diagnostic criteria of AMD into type 1 nAMD, type 2 nAMD, PCV, and RAP^2,22^. BCVA was converted to logMAR units (logMAR VA) for statistical analysis. For measurement of CRT, the ETDRS grid was employed^23^. In this study, CRT was defined as the mean retinal thicknesses of a central 1-mm circle on the ETDRS grid in the macula^65^.

### Aqueous humor sample collection and cytokine measurements

In nAMD group, before the first intravitreal injection of anti-VEGF agents, approximately 0.1 mL of undiluted aqueous humor was collected by performing an anterior chamber limbal paracentesis. In control group, undiluted aqueous humor samples were obtained at the beginning of cataract surgery. The aqueous humor samples were transferred into sterile tubes and stored at −80 °C until processing. No complication associated with the sampling of aqueous humor occurred. Twenty-seven cytokines in the aqueous humor samples were measured by a Bio-Plex multiplex assay (Bio-Plex Human Cytokine 27-plex panel; Bio-Rad, Hercules, CA, USA) and a multiplex bead analysis system (Bio-Plex Suspension Array System; Bio-Rad) according to manufacturers’ instructions. All standards and samples were assayed in duplicate. Levels of aqueous humor cytokines below detectable levels were treated as 0 for statistical analysis^9,18^.

### Statistical analysis

Statistical analyses were performed using the statistic add-in software for Excel (BellCurve for Excel®, SSRI Co., Ltd., Tokyo, Japan, and XLSTAT®, Addinsoft company, Paris, France). Data are expressed as mean ± standard deviation. Chi-squared test (for n ≥ 10), Yates’ chi-squared test (for 10 > n ≥ 5) or Fisher’s exact test (for n < 4) was used to compare categorical variables. Two-tailed Mann-Whitney *U* test and Two-tailed Spearman’s rank correlation were used for nonparametric comparison of 2 groups. Two-tailed Kruskal-Wallis test followed by post hoc two-tailed Steel–Dwass test were used for nonparametric comparison of multiple groups. Binomial logistic regression analysis was performed to examine the associations of elevated or decreased inflammatory cytokines with the pathogenesis of nAMD. In addition, PCA, EFA and hierarchical cluster analysis were used to summarize various individual factors to simple groups with similar properties. Inflammatory cytokines with over 30% detection rate in nAMD group were adopted as explanatory variables in binomial logistic regression analysis, PCA, EFA and multiple regression analysis, and those without 0% detection rate were applied in hierarchical cluster analysis. EFA was conducted as follows: (1) default value was the value of squared multiple correlation; (2) loading was extracted using maximum likelihood; (3) rotation was performed with the Promax method. Hierarchical cluster analysis was performed using Euclidean distance as a distance measure and Ward’s method for hierarchical clustering. A *P* level less than 0.05 was considered to be statistically significant.

## Supplementary information

Dataset 1

## Data Availability

The datasets analyzed and/or used in the present study are available from the corresponding author upon request.
