# Supplementary material for: Comprehensive expression patterns of inflammatory cytokines in aqueous humor of patients with neovascular age-related macular degeneration
Source: Sci Rep. 2019 Dec 19;9:19447. doi: 10.1038/s41598-019-55191-x (PMC6923359; doi:10.1038/s41598-019-55191-x)
Supplement: Supplementary file 1 — Dataset 1 [file 41598_2019_55191_MOESM1_ESM.docx]

**Comprehensive expression patterns of inflammatory cytokines in aqueous humor of patients with neovascular age-related macular degeneration**

Tomohito Sato^1^ MD, PhD, Masaru Takeuchi^1^* MD, PhD, Yoko Karasawa^1^ PhD, Kei Takayama^1^ MD, PhD and Toshio Enoki^2^ MD.

^1^Department of Ophthalmology, National Defense Medical College, Tokorozawa, Saitama, Japan

^2^Enoki Eye Clinic, Sayama, Saitama, Japan

**Corresponding Author**: Masaru Takeuchi, M.D., Ph.D.

Chairman and Professor

Department of Ophthalmology, National Defense Medical College

3-2 Namiki, Tokorozawa, Saitama, 359-8513, Japan

Tel: +81-4-2995-1683

Fax: +81-4-2993-5332

Email: masatake@ndmc.ac.jp

**Supplementary Table S1. Age, visual acuity, central retinal thickness and aqueous humor levels of cytokines stratified by gender in type 1 nAMD patients and controls.**

Four groups were comprised of type 1-male, -female nAMD groups and control-male, -female groups. Age, logMAR VA, CRT and cytokine levels were compared among 4 groups by two-tailed Kruskal-Wallis test. Cytokine values are given in units of pg/ml.

*N*; number, VA; visual acuity, CRT; central retinal thickness, *; *P* < 0.05, **; *P* < 0.01.

**Supplementary Table S2.** **Comparisons of age, visual acuity, central retinal thickness and aqueous humor levels of cytokines stratified by gender in type 1 nAMD patients and controls.**

Age, logMAR VA, CRT and cytokine levels were compared among 4 groups by Kruskal-Wallis test followed by post-hoc Steel–Dwass test for each comparison. *; *P* < 0.05, **; *P* < 0.01.
